# The heat shock protein 90 inhibitor BIIB021 suppresses the growth of T and natural killer cell lymphomas

**DOI:** 10.3389/fmicb.2015.00280

**Published:** 2015-04-09

**Authors:** Michio Suzuki, Tadashi Takeda, Hikaru Nakagawa, Seiko Iwata, Takahiro Watanabe, Mohammed N. A. Siddiquey, Fumi Goshima, Takayuki Murata, Jun-ichi Kawada, Yoshinori Ito, Seiji Kojima, Hiroshi Kimura

**Affiliations:** ^1^Department of Pediatrics, Nagoya University Graduate School of MedicineNagoya, Japan; ^2^Department of Virology, Nagoya University Graduate School of MedicineNagoya, Japan

**Keywords:** Epstein-Barr virus, LMP1, T and NK cell lymphoma, heat shock protein 90, BIIB021

## Abstract

Epstein-Barr virus (EBV), which infects not only B cells but also T and natural killer (NK) cells, is associated with a variety of lymphoid malignancies. Because EBV-associated T and NK cell lymphomas are refractory and resistant to conventional chemotherapy, there is a continuing need for new effective therapies. EBV-encoded “latent membrane protein 1” (LMP1) is a major oncogene that activates nuclear factor kappa B (NF-κB), c-Jun N-terminal kinase (JNK), and phosphatidylinositol 3-kinase signaling pathways, thus promoting cell growth and inhibiting apoptosis. Recently, we screened a library of small-molecule inhibitors and isolated heat shock protein 90 (Hsp90) inhibitors as candidate suppressors of LMP1 expression. In this study, we evaluated the effects of BIIB021, a synthetic Hsp90 inhibitor, against EBV-positive and -negative T and NK lymphoma cell lines. BIIB021 decreased the expression of LMP1 and its downstream signaling proteins, NF-κB, JNK, and Akt, in EBV-positive cell lines. Treatment with BIIB021 suppressed proliferation in multiple cell lines, although there was no difference between the EBV-positive and -negative lines. BIIB021 also induced apoptosis and arrested the cell cycle at G1 or G2. Further, it down-regulated the protein levels of CDK1, CDK2, and cyclin D3. Finally, we evaluated the *in vivo* effects of the drug; BIIB021 inhibited the growth of EBV-positive NK cell lymphomas in a murine xenograft model. These results suggest that BIIB021 has suppressive effects against T and NK lymphoma cells through the induction of apoptosis or a cell cycle arrest. Moreover, BIIB021 might help to suppress EBV-positive T or NK cell lymphomas via the down-regulation of LMP1 expression.

## Introduction

Epstein-Barr virus (EBV), which was discovered in Burkitt lymphoma cells in 1964, was the first human oncovirus to be described (Epstein et al., 1964). EBV infects B cells, and is associated with several B malignancies, including Burkitt lymphoma, Hodgkin lymphoma, and post-transplant lymphoproliferative disorders (Cohen, 2000; Kimura et al., 2013). However, EBV infects not only B cells but also T and natural killer (NK) cells and is associated with a variety of T cell and NK cell malignancies, including extranodal NK/T cell lymphoma nasal type (Chan et al., 2008b), hydroa vacciniforme-like lymphoma (Quintanilla-Martinez et al., 2008), aggressive NK cell leukemia (Chan et al., 2008a), and chronic active EBV disease (CAEBV) (Fox et al., 2011; Kimura et al., 2012, 2013). Some of these malignancies are refractory and resistant to conventional chemotherapies, partly because of their expression of P-glycoproteins (Suzuki et al., 2010). Thus, effective treatments for T and NK cell lymphomas are needed, and novel approaches involving targeted molecular therapies are desirable.

Infection with EBV has two possible outcomes. In a lytic infection, virions are produced and the host cell is lysed. Alternatively, EBV can latently infect cells by generating an episome, or circular EBV genome, in the nucleus of host lymphocytes. Latent infections may be subdivided into three types based on the pattern of EBV gene expression as follows: latency I: EBV nuclear antigen 1 (EBNA1), EBV-encoded small RNA (EBER), and *Bam*HI-A rightward transcripts (BARTs); latency II: EBNA1, EBER, BARTs, latent membrane protein 1 (LMP1), and LMP2; and latency III: EBNA1, EBER, BARTs, LMP1, LMP2, EBNA2, EBNA3, and EBNA-LP (Cohen, 2000).

LMP1 is a major oncoprotein that activates the nuclear factor kappa B (NF-κB), c-Jun N-terminal kinase (JNK), and phosphatidylinositol 3-kinase (PI3K) signaling pathways, thus inhibiting apoptosis and promoting cell growth and proliferation (Damania, 2004). LMP1 is expressed in most EBV-positive T and NK cell lymphomas because latency II is seen in these EBV-infected cells (Kubota et al., 2008; Iwata et al., 2010).

We previously screened small-molecule inhibitors that repressed LMP1 expression to explore potential targets for the treatment of T and NK cell lymphomas (Murata et al., 2013). Using EBV-positive cell lines and a library of small-molecule inhibitors, we identified radicicol and 17-allyl-17-demethoxygeldanamycin (17-AAG). Interestingly, both drugs were heat shock protein 90 (Hsp90) inhibitors.

Hsp90 is a chaperone that functions in the correct folding of its client proteins to their active conformation (Jhaveri et al., 2012). Overexpression of Hsp90 is detected in various cancers, and many of the client proteins of Hsp90 include oncoproteins with important functions in the development and promotion of cancer (Whitesell and Lindquist, 2005). Recently, some Hsp90 inhibitors have been evaluated in cancer (Jhaveri et al., 2012). We already showed that radicicol and 17-AAG suppressed LMP1 expression and cell proliferation in B, T, and NK cell lymphoma cell lines (Murata et al., 2013). However, 17-AAG, a first-generation Hsp90 inhibitor, has limitations in clinical use because of drug insolubility and hepatotoxicity, diarrhea, and fatigue (Jhaveri et al., 2012).

BIIB021, a second-generation Hsp90 inhibitor, is a purine scaffold-based Hsp90 inhibitor that binds to the ATP-binding pocket of Hsp90 and interferes with Hsp90 chaperone function, resulting in client protein degradation and tumor growth inhibition (Lundgren et al., 2009; Jhaveri and Modi, 2012). Furthermore, BIIB021 can be administered orally. BIIB021 has antitumor activity against Hodgkin lymphoma and Kaposi sarcoma-associated herpes virus-associated primary effusion lymphoma (Boll et al., 2009; Gopalakrishnan et al., 2013), and it has been evaluated in a phase I clinical trial involving solid tumors (Saif et al., 2014) and a phase II clinical trial involving gastrointestinal stromal tumors (Dickson et al., 2013). In this study, we explored the effects of the Hsp90 inhibitor BIIB021 in EBV-associated T and NK lymphoma cell lines. Additionally, we investigated the *in vivo* effects of BIIB021 in the NOD/Shi-scid/IL-2Rγ^null^ (NOG) mouse model.

## Materials and methods

### Cell lines and reagents

SNT13 and SNT16 are EBV-positive T cell lines (Zhang et al., 2003), and KAI3 (Tsuge et al., 1999) and SNK6 (Zhang et al., 2003) are EBV-positive NK cell lines. Jurkat (Kaplan et al., 1976) and KHYG1 (Yagita et al., 2000) are EBV-negative T and NK cell lines, respectively. SNT13, SNT16, and KAI3 were derived from patients with CAEBV, and SNK6 was derived from an extranodal NK/T-cell lymphoma, nasal type. MT-2/rEBV/9-7 and MT-2/rEBV/9-9 were established through the infection of MT-2 cells with the hygromycin-resistant EBV strain B-95 (Miyoshi et al., 1981; Fujiwara and Ono, 1995). MT-2/hyg/CL2 and MT-2/hyg/CL3 cells were transfected with a hygromycin resistance gene as controls. NKL was derived from a patient with large granular lymphocyte leukemia (Robertson et al., 1996), and the TL1 cell line was established from NKL cells infected with an Akata-transfected recombinant EBV strain carrying a neomycin resistance gene (Isobe et al., 2008). The characteristics of each cell line are summarized in Table 1.

**Table 1:** **Characteristics of the cell lines**.

**Cell type**	**Cell line**	**EBV**	**Cell origin**
T cell line	SNT13	+	CAEBV
	SNT16	+	CAEBV
	Jurkat	−	Acute T lymphoblastic leukemia
	MT-2/rEBV/9−7	+	MT-2 cell line (adult T-cell leukemia)
	MT-2/rEBV/9−9	+	MT-2 cell line (adult T-cell leukemia)
	MT-2/hyg/CL2	−	MT-2 cell line (adult T-cell leukemia)
	MT-2/hyg/CL3	−	MT-2 cell line (adult T-cell leukemia)
NK cell line	KAI3	+	CAEBV
	SNK6	+	Extranodal NK/T cell lymphoma
	KHYG1	−	Aggressive NK cell leukemia
	TL1	+	NKL cell line
	NKL	−	Large granular lymphocyte leukemia

*EBV, Epstein-Barr virus; CAEBV, Chronic active Epstein-Barr virus disease*.

BIIB021 was purchased from LC Laboratories (Woburn, MA). It was dissolved in dimethyl sulfoxide (DMSO).

### Real-time RT-PCR

Cells were treated with DMSO or 5 μM BIIB021 for 24 and 48 h, after which RNA was extracted using a QIAmp RNeasy Mini Kit (Qiagen, Hilden, Germany). Viral mRNA expression was quantified by one-step multiplex real-time reverse transcription (RT)-PCR using an Mx3000P real-time PCR system (Stratagene, La Jolla, CA), as described previously (Kubota et al., 2008; Iwata et al., 2010). The expression of viral mRNA was determined in comparison with β_2_-microglobulin mRNA as an endogenous control. Each experiment was conducted in duplicate; the results are shown as the mean of two samples.

### Western blotting

After treatment with DMSO or 5 μM BIIB021 for 24 and 48 h, whole-cell extracts were lysed directly with sodium dodecyl sulfate (SDS) sample buffer (50 mM Tris-HCl, pH 6.8, 2% sodium dodecyl sulfate, 10% glycerol, 6% 2-mercaptoethanol, and 0.0025% bromophenol blue), briefly sonicated, and separated by SDS polyacrylamide gel electrophoresis (SDS-PAGE). The primary antibodies used were as follows: anti-LMP1 (S12) at 1:50, anti-EBNA1 (provided by Teru Kanda, Aichi Cancer Center, Aichi, Japan) at 1:1000, anti-β-actin (#A5316; Sigma, St. Louis, MO) at 1:5000, anti-phospho-Akt (#4058; Cell Signaling Technology, Danvers, MA) at 1:1000, anti-Akt (#9272; Cell Signaling Technology) at 1:1000, anti-NF-κB (p65) (#610868; BD Biosciences, Franklin Lakes, NJ) at 1:250, anti-IκBα (#4814; Cell Signaling Technology) at 1:1000, anti-poly(ADP-ribose) polymerase (PARP) (P248; Sigma) at 1:1000, anti-CDK1 (#612306; BD Biosciences) at 1:250, anti-CDK2 (#610145; BD Biosciences) at 1:2500, anti-cyclin D3 (#610279; BD Biosciences) at 1:1000, anti-JNK (#9258; Cell Signaling Technology) at 1:1000, anti-phospho-JNK (#4668; Cell Signaling Technology), anti-janus kinase 3 (JAK3) (#3775; Cell Signaling Technology) at 1:1000, anti-signal transducer and activator of transcription (STAT) 3 (#4904; Cell Signaling Technology) at 1:1000, anti-phospho-STAT3 (#9145; Cell Signaling Technology) at 1:1000, anti-STAT5 (#9363; Cell Signaling Technology) at 1:1000, and anti-phospho-STAT5 (#4322; Cell Signaling Technology) at 1:1000. The secondary antibodies used were goat anti-mouse IgG-HRP conjugate (AMI3404; BioSource International, Camarillo, CA) and HRP-goat anti-rabbit IgG (H+L) (656120; Invitrogen, Carlsbad, CA). The bands were visualized using a WEST-one Western Blot Detection System (iNtRON Biotechnology, Seongnam, Korea) or Chemi-Lumi One Super (Nacalai Tesque, Kyoto, Japan). To compare the amount of each protein, densitometric analysis was performed using ImageJ software version 1.46r (NIH, Bethesda, MD).

### Cell viability assay

Cells (2 × 10^5^/mL) were cultured in 24-well plates for 96 h with DMSO or various concentrations of BIIB021, and cell viability was quantified by trypan blue exclusion. Human peripheral blood mononuclear cells (PBMCs) were isolated from healthy donors using Ficoll-Paque gradient centrifugation and cultured in 24-well plates for 96 h with 5 μM BIIB021 or DMSO. These experiments were performed in triplicate; the results shown are the mean of three wells.

### Flow cytometric apoptosis assay

Cells (2 × 10^5^/mL) were cultured in 24-well plates for 48 h with DMSO or 5 μM BIIB021, and apoptosis was measured by flow cytometry using an annexin V-phycoerythrin (PE)/7-aminoactinomycin D (7-AAD) apoptosis assay kit (BD Pharmingen Biosciences, San Diego, CA) according to the manufacturer's protocol. Stained cells were analyzed using a FACSCalibur (Becton Dickinson, San Jose, CA) flow cytometer and FlowJo (Tree Star, Ashland, OR) software.

### Cell cycle assay

Cells (2 × 10^5^/mL) were cultured in 24-well plates for 48 h with DMSO or 5 μM BIIB021, fixed with 70% ethanol, and then washed with ice-cold PBS. The fixed cells were treated with DNase-free RNase and stained with propidium iodide (Sigma). The stained cells were analyzed with a FACSCalibur flow cytometer and ModFit LT software (Verity Software House, Topsham, ME). These experiments were performed in triplicate; the results shown are the mean of three wells.

### Animal studies

Female NOG mice were purchased from the Central Institute for Experimental Animals, Kawasaki, Japan. SNK6 cells (1 × 10^6^ cells) were injected subcutaneously into the right flank of the six mice on day 0, as described previously (Siddiquey et al., 2014). DMSO or BIIB021 (120 mg/kg) was administered orally to three mice each three times per week, from day 4 to day 30. The dimensions of the tumors were measured using calipers twice per week and calculated using the formula: π × short axis × long axis × height/6. The student's *t*-test was used to compare tumor volumes. All animal experiments were approved by the University Committee in accordance with the Guidelines for Animal Experimentation at Nagoya University (Nagoya, Japan).

## Results

### BIIB021 decreases LMP1 gene and protein expression in EBV-positive T and NK cell lines

**Figure 1:**
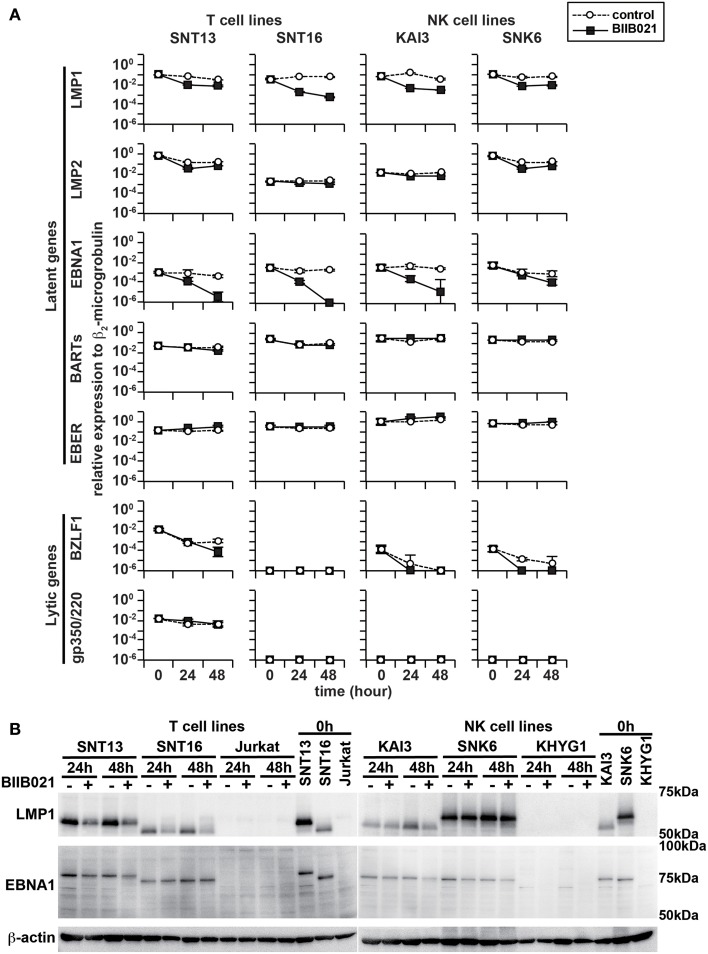
**BIIB021 decreased the expression of latent membrane protein 1 (LMP1) and Epstein-Barr virus (EBV) nuclear antigen 1 (EBNA1). (A)** EBV-positive T cell lines (SNT13 and SNT16) and EBV-positive NK cell lines (KAI3 and SNK6) were treated with DMSO or 5 μM BIIB021 and harvested after 0, 24, and 48 h to evaluate EBV-encoded gene expression using real-time RT-PCR. β_2_-Microglobulin was used as an endogenous control and reference gene for relative quantification and assigned an arbitrary value of 1 (10°). All data are expressed as means ± SEM. **(B)** Cells were treated with DMSO or 5 μM BIIB021 for 24 or 48 h. Cell lysates were collected and immunoblotting was performed using anti-LMP1 and -EBNA1 antibodies. β-Actin was used as a loading control.

First, we examined the effects of BIIB021 on LMP1 gene and protein expression in EBV-positive cell lines. Figure 1A shows the quantification of EBV mRNA expression by real-time RT-PCR. BIIB021 decreased LMP1 gene expression in all EBV-positive cell lines. Next, we confirmed the expression of LMP1 protein by Western blotting (Figure 1B and Supplementary Table 1). Although the expression levels of LMP1 differed among cell lines, probably due to the difference of regulation of LMP1 in each cell line, BIIB021 decreased the expression of LMP1. We also examined the expression of other EBV-encoded latent and lytic genes (Figure 1A). BIIB021 decreased EBNA1 gene expression in all EBV-positive cell lines and decreased EBNA1 protein expression in some cell lines (Figure 1B and Supplementary Table 1). However, BIIB021 did not affect the expression of other latent genes, although it slightly decreased BZLF1 mRNA in some cell lines (Figure 1A).

### BIIB021 decreased the viability of T and NK cell lines

**Figure 2:**
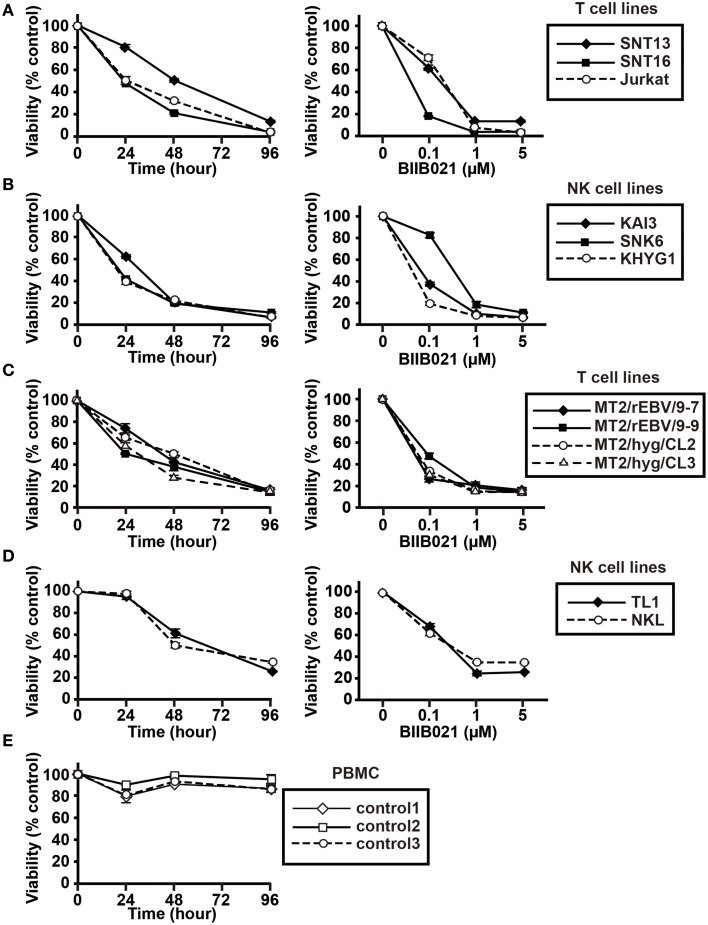
**BIIB021 decreased the viability of EBV-positive and -negative T and NK cells and showed no adverse effects on human PBMCs. (A)** EBV-positive (SNT13 and SNT16) and -negative (Jurkat) T cell lines, **(B)** EBV-positive (KAI3 and SNK6) and -negative (KHYG1) NK cell lines, **(C)** EBV-positive T cell lines (MT2/rEBV/9-7 and MT2/rEBV/9-9) and EBV-negative T cell lines (MT2/hyg/CL2 and MT2/hyg/CL3), **(D)** an EBV-positive NK cell line (TL1), and an EBV-negative NK cell line (NKL) were used. Each cell line was cultured with DMSO or various concentrations of BIIB021. Viability was calculated as the percentage of viable cells among BIIB021-treated cells vs. DMSO-treated cells. Each cell line was cultured with 5 μM BIIB021 for the indicated times (left) or with the indicated concentrations of BIIB021 for 96 h (right). The filled markers represent EBV-positive cell lines and the open markers represent EBV-negative cell lines. **(E)** Human PMBCs, isolated from three healthy volunteers, were cultured with DMSO or 5 μM BIIB021 for 96 h. Viability was calculated as the percentage of viable cells among BIIB021-treated cells vs. DMSO-treated cells. Bars indicate SEMs.

We next evaluated the effects of BIIB021 on the viability of T and NK cell lines (Figure 2). EBV-positive T cell lines (SNT13 and SNT16) and NK cell lines (KAI3 and SNK6) were cultured with various concentrations of BIIB021. BIIB021 decreased the viability of the EBV-positive T and NK cell lines in a time- and dose-dependent manner. In comparison, when the EBV-negative T cell line (Jurkat) and NK cell line (KHYG1) were cultured under the same conditions, BIIB021 decreased their viability. Indeed, we found no significant differences between the EBV-positive and -negative cell lines (Figures 2A,B). To directly compare the effects of BIIB021 on EBV-positive and -negative cells, we administered BIIB021 to EBV-infected MT2 cell lines and their controls (T cell lines, Figure 2C), TL1 and parental NKL cells (NK cell lines, Figure 2D). We found no difference in the effects of BIIB021 on these cell lines. Moreover, we administered BIIB021 to human PBMCs to evaluate any adverse effects. More than 80% of the cells were viable after 96 h (Figure 2E), indicating modest toxicity.

### BIIB021 induces apoptosis in T and NK cell lines

**Figure 3:**
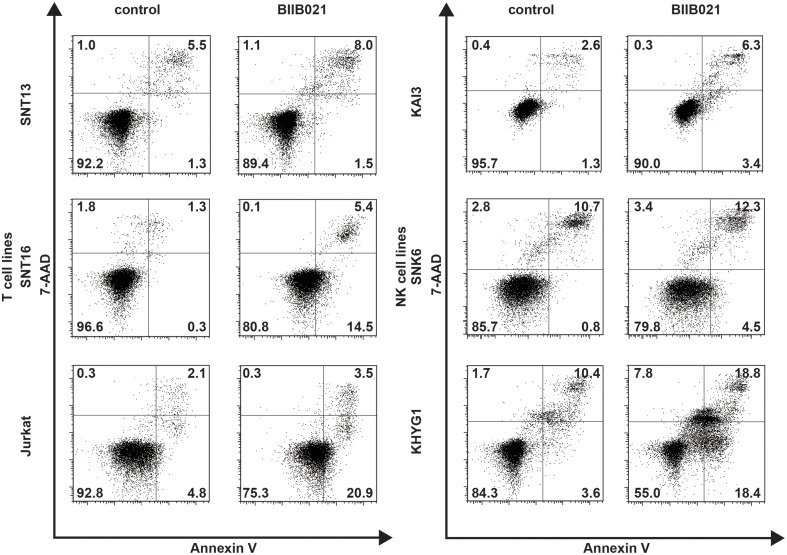
**BIIB021 induced apoptosis in some EBV-positive and -negative T and NK cell lines**. EBV-positive T cell lines (SNT13 and SNT16), an EBV-negative T cell line (Jurkat), EBV-positive NK cell lines (KAI3 and SNK6), and an EBV-negative NK cell line (KHYG1) were cultured with DMSO or 5 μM BIIB021 for 48 h. Apoptosis was measured by flow cytometry after annexin V-phycoerythrin (annexin V) and 7-aminoactinomycin D (7-AAD) staining.

**Figure 4:**
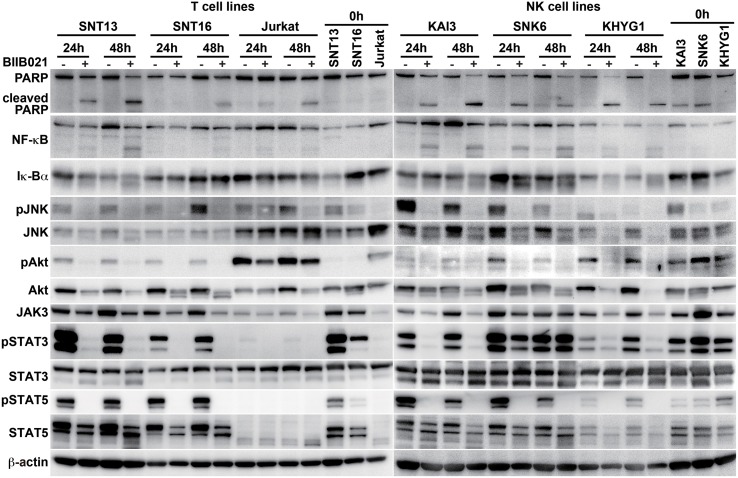
**BIIB021 decreased apoptosis- and cell growth-related protein expression in EBV-positive and -negative cell lines**. EBV-positive T cell lines (SNT13 and SNT16), an EBV-negative T cell line (Jurkat), EBV-positive NK cell lines (KAI3 and SNK6), and an EBV-negative NK cell line (KHYG1) were cultured with DMSO or 5 μM BIIB021 for 24 or 48 h. Whole cell lysates were collected and subjected to immunoblotting using anti-poly(ADP-ribose) polymerase (PARP), -nuclear factor kappa B (NF-κB), -Iκ-Bα, -c-Jun N-terminal kinase (JNK), -phospho-JNK, -Akt, -phospho Akt, -Janus kinase 3 (JAK3), -signal transducer and activator of transcription (STAT) 3, -phospho-STAT3, -STAT5, phospho-STAT5, and -β-actin antibodies.

Next, we analyzed the induction of apoptosis by flow cytometry with annexin V and 7-AAD staining. BIIB021 increased early apoptotic cells, defined as those positive for annexin V and negative for 7-AAD staining, in the two T cell lines, SNT16 and Jurkat, and two NK cell lines, SNK6 and KHYG1 (Figure 3). We then investigated the levels of PARP and cleaved PARP by Western blotting. BIIB021 induced cleaved PARP in all cell lines, indicating that the drug induced apoptosis in these cells (Figure 4 and Supplementary Table 2).

### BIIB021 decreases LMP1 downstream signaling pathways in T and NK cell lines

The NF-κB, JNK, and PI3K signaling pathways are downstream of LMP1 (Young and Rickinson, 2004). Thus, we examined the expression of these proteins (Figure 4 and Supplementary Table 2). BIIB021 decreased NF-κB, JNK, phospho-JNK, Akt, and phospho-Akt expression, although the extent of the decrease differed among the cell lines. However, BIIB021 also decreased the expression of these proteins in EBV-negative cells. We also examined the JAK/STAT pathway that is presumably upstream of LMP1. BIIB021 decreased JAK3, phospho-STAT3, and phospho-STAT5, indicating that the drug inhibits the JAK/STAT pathway (Figure 4 and Supplementary Tables 2, 3).

### BIIB021 induces a cell cycle arrest in T cell and NK cell lines

**Figure 5:**
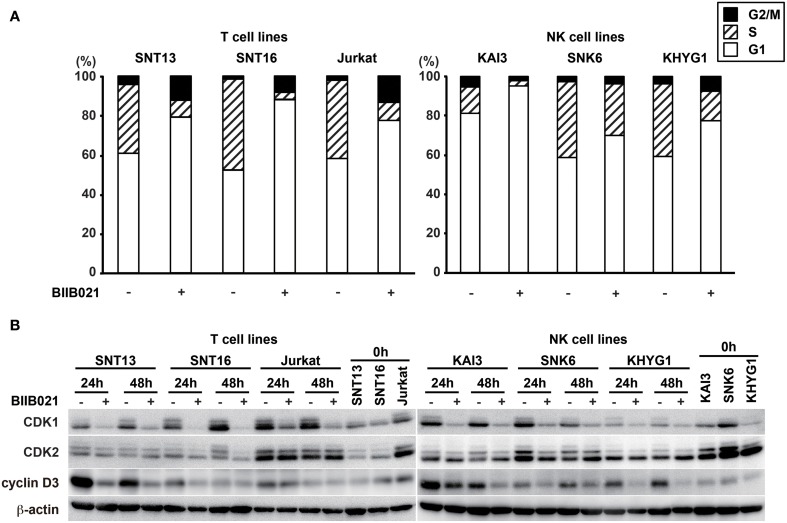
**BIIB021 induced a cell cycle arrest in EBV-positive and -negative cell lines. (A)** EBV-positive T cell lines (SNT13 and SNT16), an EBV-negative T cell line (Jurkat), EBV-positive NK cell lines (KAI3 and SNK6), and an EBV-negative NK cell line (KHYG1) were cultured with DMSO or 5 μM BIIB021 for 48 h. Cell cycle status was measured by flow cytometry using propidium iodide staining. **(B)** Cells were cultured with DMSO or 5 μM BIIB021 for 24 or 48 h. Whole cell lysates were collected and subjected to immunoblotting using anti-CDK1, -CDK2, -cyclin D3, and -β-actin antibodies.

To determine the mechanism of the growth inhibition induced by BIIB021, we investigated the effects of BIIB021 on the cell cycle using flow cytometry with propidium iodide staining (Figure 5A). The population of cells in both G1 and G2 increased in all T cell lines following exposure to BIIB021, while the population in G1 phase increased in the NK cell lines. Several cell cycle-related proteins were investigated by Western blotting (Figure 5B and Supplementary Table 4). CDK1, which is associated with the G2/M checkpoint, and CDK2 and cyclin D3, which are associated with the G1 checkpoint, were decreased by BIIB021 in all of the cell lines tested.

### BIIB021 inhibits EBV-positive NK cell lymphoma growth in a murine xenograft model

**Figure 6:**
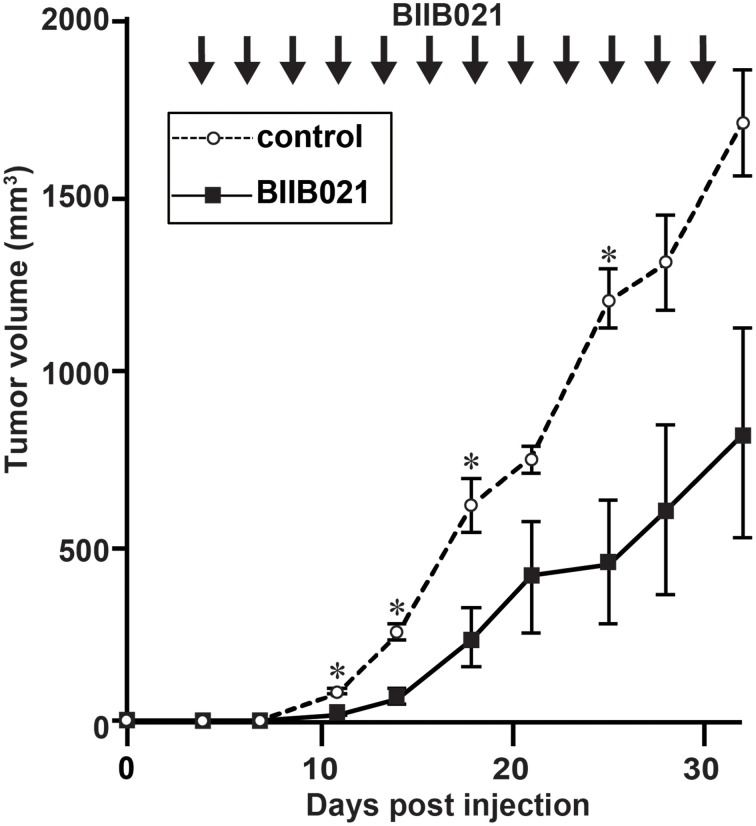
**BIIB021 inhibited the growth of EBV-positive NK cell lymphomas in a murine xenograft model**. NOD/Shi-scid/IL-2Rγ^null^ (NOG) mice were implanted subcutaneously with 1 × 10^6^ SNK6 (EBV-positive NK) cells on the right flank. From days 4 to 30, DMSO (vehicle) or BIIB021 (120 mg/kg) was administered orally three times per week. The tumor volume was measured twice per week. Bars indicate the SEM. *p < 0.05.

Finally, we investigated the effect of BIIB021 using an NOG mouse xenograft model (Figure 6). NOG mice are completely immunodeficient and can accept the EBV-positive NK cell line SNK6 (Siddiquey et al., 2014). When we inoculated SNK6 cells subcutaneously, tumors developed rapidly. After the subcutaneous inoculation of SNK6, we administered BIIB021 orally and measured the tumor volumes. BIIB021 inhibited the growth of EBV-positive NK cell lymphomas compared with the results in control mice (*p* < 0.05).

## Discussion

EBV-associated T and NK cell lymphomas are refractory and resistant to chemotherapy; thus, new treatment agents are necessary. We have shown the antitumor activities of several drugs against EBV-associated T and NK cell lymphomas (Iwata et al., 2011, 2012; Kanazawa et al., 2014; Kawada et al., 2014; Siddiquey et al., 2014). In the present study, we focused on the major oncogene LMP1, and found that the Hsp90 inhibitor BIIB021 decreased LMP1 gene expression and inhibited cell proliferation. The mechanism underlying the down-regulation of LMP1 by BIIB021 is, however, unclear.

The regulation of LMP1 expression differs between latency II and III. LMP1 transcription is regulated by EBNA2 in latency III (Laux et al., 1994; Johannsen et al., 1995), whereas in latency II, where EBV-infected T or NK cells belong, LMP1 expression is regulated by the JAK/STAT pathway, which is stimulated by cytokines, including IL-4, IL-6, IL-10, IL-13, and IL-21 (Chen et al., 2001, 2003; Kis et al., 2006, 2010, 2011). JAK/STAT signaling can be inhibited by Hsp90 inhibitors (Schoof et al., 2009). Because EBV-positive T and NK cell lines are dependent on IL-2 and LMP1 is upregulated by IL-2 (Takahara et al., 2006), Hsp90 inhibitors may suppress LMP1 expression by blocking the JAK/STAT pathway activated by IL-2 (Murata et al., 2013). In the present study, the JAK/STAT pathway was actually down-regulated byBIIB021. Moreover, JNK and NF-κB signaling, which was down-regulated by BIIB021 in this study, regulates LMP1 expression (Goormachtigh et al., 2006). BIIB021 may decrease LMP1 expression in EBV-positive T and NK cell lines in type II latency by down-regulating more than one pathway. However, Sun et al. reported that Hsp90 inhibitors increased LMP1 expression in type III latency B cell lines (Sun et al., 2010). Thus, different regulatory mechanisms of LMP1 expression may exist between type II and type III latency cells.

We suggest that BIIB021 inhibited the function of proteins upstream of LMP1, leading to the down-regulation of LMP1 and inhibition of cell growth. To clarify the role of LMP1 in the inhibitory effect of BIIB021, we attempted to silence LMP1 using siRNA in Hsp90 inhibitor-treated cells. However, because of poor induction efficiency in the EBV-positive T and NK cell lines, we have not yet succeeded. Re-induction of LMP1 in BIIB021-treated T or NK cell lines was also tried, but it was so stressful to the cell lines that we could not evaluate their cell growth and viability. We have established EBV-negative T cell lines that express LMP1 inducibly using the Tet-on system (Ito et al., 2014); however, LMP1 alone was not sufficient to enhance the proliferation of these cell lines, so they could not be used to evaluate the effect of Hsp90 inhibitors. In this study, BIIB021 also suppressed the proliferation of EBV-negative cell lines. More than 200 proteins have been reported to be Hsp90 clients (Hong et al., 2013). BIIB021 presumably inhibits the functions of many Hsp90 substrates in EBV-positive and -negative T and NK cell lines, resulting in cell growth inhibition, cell cycle arrest, and apoptosis induction. Of these mechanism, cell cycle arrest is particularly important, since BIIB021 induced the cell cycle arrest in all cell lines. Both CDK1 and CDK2 are known to be client proteins for HSP90 (Prince et al., 2005; Wang et al., 2011). They are protein kinases that regulates cell cycle progression through the association with cyclins. Activated CDK1 and CDK2 are able to phosphorylate downstream effector proteins and drive G2/M and S/G2 transitions, respectively (Malumbres and Barbacid, 2009). In this study, CDK1 and CDK2 were decreased by BIIB021 and the cell cycle was arrested at either G1 or G2 phase. Thus, BIIB021 inhibits the functions of key proteins in cell cycle and thereby causes cell growth inhibition.

BIIB021 decreased not only LMP1 but also EBNA1 expression. Sun et al. reported that Hsp90 inhibitors decreased EBNA1 expression in a latency III B cell line (Sun et al., 2010). EBNA1, which is expressed on virtually all EBV-infected cells, acts in the maintenance of the episomal EBV genome in host cells and interacts with certain viral promoters (Young and Rickinson, 2004). Additionally, EBNA1 may contribute to tumorigenesis by inhibiting apoptosis (Kennedy et al., 2003; Saridakis et al., 2005). The down-regulation of EBNA1 may also be associated with the suppression of cell growth in EBV-positive T and NK cells. Furthermore, given that EBNA1 is required for maintaining EBV episome in infected cells, EBNA1 down-regulation might lead to a loss of EBV genome, resulting in “cure” of lymphoma cells.

When we applied BIIB021 to human PBMCs, the viability of the treated cells remained high, indicating the absence of major toxic effects. Moreover, we did not observe any significant adverse events, such as body weight loss or death, in the mouse study. A phase II clinical trial of BIIB021 for gastrointestinal stromal tumors has been performed (Dickson et al., 2013). Some adverse events were observed, including nausea, vomiting, diarrhea, and hepatotoxicity; however, these effects were generally mild to moderate, so we believe that BIIB021 is likely acceptable for clinical use. Furthermore, while resistance to Hsp90 inhibitors has not been reported in clinical trials or *in vitro* experiments, drug resistance may occur with long-term use. Additionally, the effects of Hsp90 inhibitors are generally modest and are not effective enough to induce complete remission. Thus, we need combined therapies with chemotherapeutic agents or other targeted molecular therapies for the treatment of T and NK cell lymphomas.

In this paper, we showed that BIIB021 decreased LMP1 and EBNA1 mRNA and protein expression in EBV-positive T and NK cell lines. In addition, we showed that BIIB021 treatment induced apoptosis, arrested the cell cycle, and suppressed the proliferation of EBV-positive and -negative T and NK cell lines. BIIB021 also inhibited the growth of EBV-positive NK cell lymphomas in a murine xenograft model. These results suggest that BIIB021 has potential as a new treatment for EBV-associated T and NK cell lymphomas, although its effect may be independent of EBV.

## Financial support

This study was supported by grants from the Ministry of Education, Culture, Sports, Science and Technology of Japan (25293109) and from the Ministry of Health, Labour and Welfare of Japan (H26-Nanchi-013).

### Conflict of interest statement

The authors declare that the research was conducted in the absence of any commercial or financial relationships that could be construed as a potential conflict of interest.
