# Supplementary material for: The heat shock protein 90 inhibitor BIIB021 suppresses the growth of T and natural killer cell lymphomas
Source: Front Microbiol. 2015 Apr 9;6:280. doi: 10.3389/fmicb.2015.00280 (PMC4391044; doi:10.3389/fmicb.2015.00280)
Supplement: Supplementary file 1 [file SupplementaryTables1-4.PDF]

SUPPLEMENTARY TABLE 1.

Ratios of EBV-associated proteins to β-actin measured by densitometric analysis of immunoblots.

	SNT13				SNT16				Jurkat				SNT13	SNT16	Jurkat
Time (h)	24	24	48	48	24	24	48	48	24	24	48	48	0	0	0
BIIB021	-	+	-	+	-	+	-	+	-	+	-	+			
LMP1/β-actin	1.26	0.89	1.16	0.74	0.68	0.45	0.79	0.38	0.00	0.00	0.01	0.03	1.46	0.87	0.00
EBNA/β-actin	0.58	0.48	0.36	0.27	0.25	0.39	0.56	0.49	0.00	0.00	0.02	0.16	0.60	0.65	0.05

	KAI3				SNK6				KHYG1				KAI3	SNK6	KHYG1
Time (h)	24	24	48	48	24	24	48	48	24	24	48	48	0	0	0
BIIB021	-	+	-	+	-	+	-	+	-	+	-	+			
LMP1/β-actin	0.40	0.38	0.58	0.39	0.88	0.90	1.07	1.15	0.06	0.03	0.03	0.02	0.40	0.77	0.00
EBNA/β-actin	0.43	0.30	0.23	0.18	0.30	0.20	0.19	0.16	0.03	0.03	0.06	0.07	0.26	0.22	0.00

LMP1, latent membrane protein 1; EBNA1, Epstein-Barr virus nuclear antigen 1.

SUPPLEMENTARY TABLE 2.

Ratios of apoptosis- and cell growth-related proteins to β-actin measured by densitometric analysis of immunoblots.

	SNT13				SNT16				Jurkat				SNT13	SNT16	Jurkat
Time (h)	24	24	48	48	24	24	48	48	24	24	48	48	0	0	0
BIIB021	-	+	-	+	-	+	-	+	-	+	-	+			
Cleaved PARP /β-actin	0.15	0.28	0.19	0.47	0.10	0.06	0.08	0.21	0.04	0.10	0.01	0.24	0.07	0.01	0.00
NF-κB/β-actin	0.40	0.34	0.61	0.38	0.47	0.31	0.51	0.45	0.46	0.72	0.68	0.38	0.13	0.20	0.61
Iκ-Bα/β-actin	0.69	0.45	0.35	0.35	0.80	0.74	0.89	1.01	1.01	0.87	0.84	0.65	0.34	1.08	1.17
JNK/β-actin	0.24	0.13	0.26	0.11	0.21	0.15	0.22	0.20	0.64	0.84	0.91	0.98	0.46	0.62	1.40
Akt/β-actin	0.75	0.25	0.49	0.16	1.09	0.47	0.83	0.34	0.34	0.39	0.78	0.28	0.45	0.81	0.71
JAK3/β-actin	0.63	0.44	0.73	0.47	0.77	0.42	0.71	0.27	0.21	0.17	0.25	0.05	0.59	0.54	0.04
STAT3/β-actin	0.67	0.58	0.58	0.58	0.59	0.76	0.77	0.61	0.73	0.90	1.00	0.88	0.71	0.77	1.03
STAT5/β-actin	0.97	0.62	0.92	0.56	1.16	0.45	1.03	0.64	0.04	0.04	0.01	0.00	0.68	0.39	0.00

	KAI3				SNK6				KHYG1				KAI3	SNK6	KHYG1
Time (h)	24	24	48	48	24	24	48	48	24	24	48	48	0	0	0
BIIB021	-	+	-	+	-	+	-	+	-	+	-	+			
Cleaved PARP /β-actin	0.16	0.35	0.10	0.52	0.14	0.40	0.37	0.37	0.10	0.34	0.11	0.47	0.26	0.37	0.22
NF-κB/β-actin	0.44	1.01	1.21	0.58	0.39	0.37	0.64	0.39	0.31	0.06	0.25	0.19	0.21	0.04	0.04
Iκ-Bα/β-actin	0.57	0.98	0.88	0.55	1.15	1.03	1.07	0.83	0.63	0.46	0.61	0.65	0.83	0.87	0.48
JNK/β-actin	0.73	0.75	1.23	0.51	0.96	0.47	0.93	0.41	0.44	0.14	0.81	0.03	0.50	0.53	0.45
Akt/β-actin	0.68	0.73	0.76	0.42	1.09	0.73	0.76	0.44	1.10	0.42	1.78	0.07	0.54	0.51	0.54
JAK3/β-actin	0.27	0.12	0.81	0.17	0.82	0.57	0.75	0.69	0.65	0.44	1.08	0.31	0.53	0.98	0.57
STAT3/β-actin	0.82	1.19	1.11	1.19	1.04	1.30	1.29	1.07	1.15	1.89	1.88	1.58	0.89	0.89	0.90
STAT5/β-actin	0.70	0.90	1.04	0.46	0.86	0.65	1.02	0.78	0.45	0.25	0.62	0.25	0.62	0.57	0.36

PARP, poly (ADP-ribose) polymerase; NF-κB, nuclear factor kappa B; JNK, c-Jun N-terminal kinase; JAK3, janus kinase 3; STAT, signal transducer and activator of transcription.

SUPPLEMENTARY TABLE 3.

Ratios of phosphorylated proteins to total proteins measured by densitometric analysis of immunoblots.

	SNT13				SNT16				Jurkat				SNT13	SNT16	Jurkat
Time (h)	24	24	48	48	24	24	48	48	24	24	48	48	0	0	0
BIIB021	-	+	-	+	-	+	-	+	-	+	-	+			
pJNK/JNK	1.12	0.79	1.25	1.74	2.02	1.74	3.73	1.85	0.73	0.46	0.70	0.13	0.76	0.19	0.00
pAkt/Akt	0.35	0.19	0.37	0.29	0.17	0.03	0.20	0.08	3.02	1.65	1.45	3.10	0.06	0.03	0.72
pSTAT3/STAT3	1.64	0.14	1.64	0.14	1.60	0.07	1.22	0.12	0.12	0.04	0.09	0.05	1.55	1.01	0.00
pSTAT5/STAT5	0.78	0.02	0.74	0.03	1.08	0.02	1.00	0.02	0.00	0.00	0.00	0.00	0.68	0.25	0.00

	KAI3				SNK6				KHYG1				KAI3	SNK6	KHYG1
Time (h)	24	24	48	48	24	24	48	48	24	24	48	48	0	0	0
BIIB021	-	+	-	+	-	+	-	+	-	+	-	+			
pJNK/JNK	1.60	0.34	0.93	0.39	0.85	0.27	0.52	0.34	0.52	0.60	0.18	0.94	0.96	0.15	0.21
pAkt/Akt	0.34	0.23	0.31	0.29	0.43	0.00	0.10	0.08	0.62	1.47	0.78	5.06	1.20	1.76	1.44
pSTAT3/STAT3	0.83	0.00	1.02	0.05	1.20	0.78	1.09	1.24	0.42	0.14	0.75	0.09	0.83	1.15	0.97
pSTAT5/STAT5	1.58	0.20	0.89	0.47	1.36	0.32	0.81	0.17	0.55	0.19	0.92	0.00	0.11	0.23	1.26

JNK, c-Jun N-terminal kinase; STAT, signal transducer and activator of transcription.

SUPPLEMENTARY TABLE 4.

Ratios of cell cycle associated proteins to β-actin measured by densitometric analysis of immunoblots.

	SNT13				SNT16				Jurkat				SNT13	SNT16	Jurkat
Time (h)	24	24	48	48	24	24	48	48	24	24	48	48	0	0	0
BIIB021	-	+	-	+	-	+	-	+	-	+	-	+			
CDK1/β-actin	0.46	0.12	0.56	0.21	0.71	0.10	1.05	0.08	1.32	0.72	1.31	0.38	0.43	0.34	0.69
CDK2/β-actin	0.59	0.41	0.36	0.46	0.49	0.40	0.52	0.22	1.31	1.44	1.15	1.19	0.25	0.38	1.15
Cyclin D3/β-actin	1.50	0.53	1.00	0.46	0.63	0.39	0.37	0.31	0.87	0.81	0.39	0.32	0.24	0.55	0.64

	KAI3				SNK6				KHYG1				KAI3	SNK6	KHYG1
Time (h)	24	24	48	48	24	24	48	48	24	24	48	48	0	0	0
BIIB021	-	+	-	+	-	+	-	+	-	+	-	+			
CDK1/β-actin	0.63	0.12	0.47	0.14	0.65	0.19	0.35	0.15	0.22	0.06	0.17	0.06	0.22	0.47	0.16
CDK2/β-actin	0.61	0.47	0.42	0.48	1.04	0.68	0.62	0.96	0.75	0.64	0.49	0.65	0.85	1.19	1.17
Cyclin D3/β-actin	0.96	0.66	0.58	0.32	0.40	0.32	0.54	0.53	0.82	0.32	0.65	0.18	0.20	0.22	0.09

CDK, cyclin-dependent kinase.
